# Bioactive Compounds, Antioxidant Capacity and Mineral Profile of Pulp and Peel from Diverse *Selenicereus* (Pitaya) Varieties in Brazil

**DOI:** 10.1007/s11130-026-01553-6

**Published:** 2026-07-30

**Authors:** Rogerio Lopes Vieites, Andres Felipe Gaona Acevedo

**Affiliations:** Department of Horticulture, Universidade Estadual Paulista (UNESP), Botucatu, Brazil

**Keywords:** Pitaya varieties, Nutritional composition, Phenolic compounds, Betalains, Functional foods

## Abstract

Pitaya (dragon fruit) has gained increasing commercial interest due to its nutritional value and bioactive potential. This study characterized seven pitaya varieties (‘Costa Rica’, ‘Nicarágua’, ‘Purple Haze’, ‘Physical Graffiti’, ‘Imperial’, ‘Connie Mayer’, and ‘Golden de Israel’) regarding physicochemical attributes of the pulp and biochemical and mineral composition of peel and pulp tissues. Physicochemical parameters, color attributes, betalains, flavonoids, total phenolic compounds, antioxidant activity (DPPH, ABTS, and FRAP), and mineral nutrients were evaluated, followed by principal component and hierarchical cluster analyses. Red-fleshed varieties (‘Costa Rica’ and ‘Nicarágua’) exhibited higher betalain contents, whereas purple-fleshed varieties (‘Purple Haze’ and ‘Physical Graffiti’) showed greater phenolic and flavonoid concentrations and higher antioxidant activity. Both peel and pulp showed favorable mineral profiles, particularly for potassium and iron. ‘Physical Graffiti’ presented the highest mineral macronutrient levels, while ‘Connie Mayer’ exhibited the highest micronutrient contents. These results highlight the nutritional and functional potential of pitaya fruits and their by-products for food applications.

**Supplementary Information:**

The online version contains supplementary material available at https://doi.org/10.1007/s11130-026-01553-6.

## Introduction

In recent years, fruits from the family *Cactaceae*, subfamily *Cactoideae*, have attracted increasing scientific and commercial interest due to their high nutritional value and richness in bioactive compounds. Among them, pitaya (*Selenicereus* spp.), also known as dragon fruit, has gained prominence as a functional food with growing global demand. Native to Mexico and Central South America, pitaya is currently cultivated in several tropical and subtropical regions worldwide [1]. Advances in molecular phylogeny have led to taxonomic reclassification, consolidating species formerly assigned to *Hylocereus* within the genus *Selenicereus*, which now encompasses most commercially cultivated pitaya varieties [2]. Pitaya fruits are recognized as an important source of bioactive compounds derived from different metabolic pathways, particularly phenolic compounds and nitrogen-containing pigments such as betalains. These metabolites are synthesized by plants as protective responses to biotic and abiotic stresses and, when consumed, exert significant antioxidant activity in the human health [3]. Experimental evidence, including in vivo studies, has demonstrated that pitaya fruits exhibit a wide range of biological activities, including antimicrobial, hepatoprotective, hypoglycemic, wound-healing, antiproliferative, and antinociceptive effects [4]. Such properties support their potential role in the prevention and management of metabolic disorders such as diabetes, obesity, and liver diseases, as well as in immune system modulation [5]. Additionally, pitaya fruits contribute significantly to dietary vitamin C intake, reinforcing their nutritional and functional relevance [6].

Beyond fresh consumption, pitaya fruits represent an attractive raw material for the food industry, particularly due to the high proportion of peel, which can account for approximately 30% of the fruit mass. This byproduct is a promising source of pectin, with reported yields ranging from 15 to 30%, as well as natural colorants such as betalains, including red betacyanins and yellow betaxanthins [7]. The growing interest in clean-label ingredients and natural antioxidants has intensified research on the valorization of fruit byproducts, positioning pitaya peel as a potential functional ingredient for food applications. Although the number of studies on pitaya has increased substantially in recent years [8], most investigations have primarily focused on the edible pulp, with limited attention given to the peel. This imbalance is largely due to traditional consumption patterns and the predominance of pulp-based food formulations [6]. However, considering the high market value of pitaya fruits and the significant volume of peel generated during processing, the characterization of peel bioactive and nutritional properties is essential for promoting sustainable use and reducing food waste. Moreover, several commercially promising pitaya varieties, including ‘Costa Rica’, ‘Nicarágua’, ‘Purple Haze’, ‘Physical Graffiti’, ‘Imperial’, ‘Connie Mayer’, and ‘Golden de Israel’, have attracted increasing attention due to their distinct fruit coloration, bioactive composition, and agronomic performance. However, comparative information regarding their physicochemical, nutritional, and functional attributes remains limited, highlighting the need for comprehensive characterization studies [9].

Despite existing reports on the physicochemical and bioactive composition of pitaya fruits, comparative studies evaluating both pulp and peel across multiple commercially relevant varieties remain scarce. Most studies focus on species-level characterization, overlooking the substantial variability that may occur among varieties within the same species. A comprehensive assessment of bioactive compounds, antioxidant capacity, and mineral composition in different fruit tissues is therefore necessary to better understand their functional potential and to support targeted applications in food systems. In this context, the present study aimed to characterize seven pitaya varieties, ‘Costa Rica’, ‘Nicarágua’, ‘Purple Haze’, ‘Physical Graffiti’, ‘Imperial’, ‘Connie Mayer’, and ‘Golden de Israel’—with respect to the physicochemical properties of the pulp and the biochemical and nutritional attributes of both pulp and peel. By integrating conventional analyses with multivariate statistical approaches, this work provides a detailed comparative overview of quality attributes and bioactive potential, contributing to the valorization of pitaya fruits and their byproducts for food and functional ingredient applications.

## Materials and Methods

The “Materials and Methods” section is available in the Supplementary information.

## Results and Discussion

The analysis of variance (ANOVA) for the factor pitaya fruit varieties ‘Costa Rica’ (CR), ‘Nicarágua’ (N), ‘Purple Haze’ (PH), ‘Physical Graffiti’ (PG), ‘Imperial’ (I), ‘Connie Mayer’ (CM), and ‘Golden de Israel’ (G), the factor fruit parts (peel and pulp), and the interaction between these factors showed significant differences for all evaluated parameters (Supplementary Table 1).

### Physicochemical Attributes of Pitaya Pulp

**Fig. 1:**
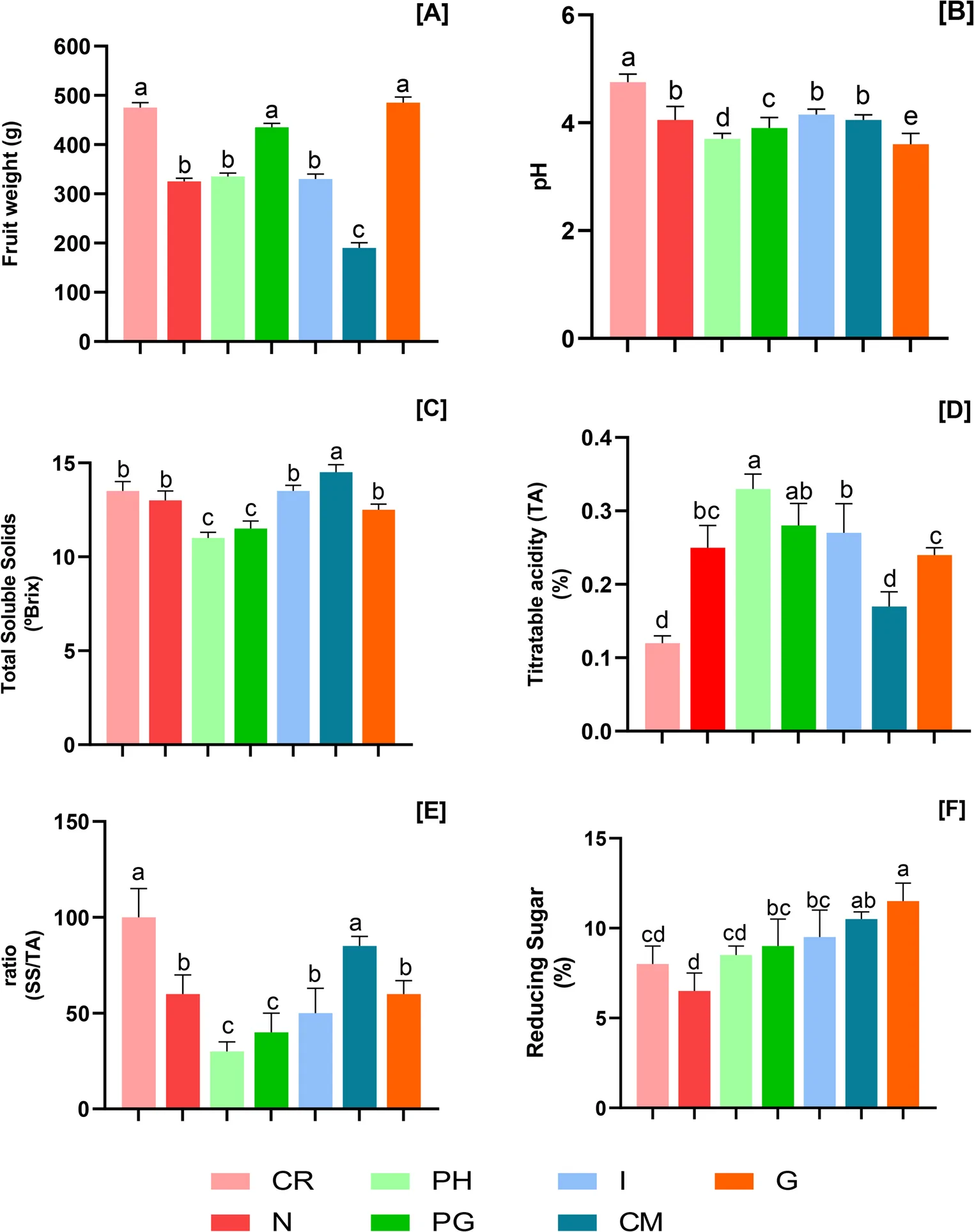
Physical-chemical parameters. Fruit weight (g) [**A**], pH [**B**], Total soluble solids (ºBrix) [**C**], Titratable acidity (% malic acid) [**D**], Ratio (TSS/TA) [E], Reducing sugar (%) [**F**] of the pulp of pitaya fruits of the varieties ‘Costa Rica’ (CR), ‘Nicaragua’ (N), ‘Purple Haze’ (PH), ‘Physical Graffiti’ (PG), ‘Imperial’ (I), ‘Connie Mayer’ (CM), and ‘Golden de Israel’ (G). Letters above the bars indicate varieties that differ significantly according to the LSD test (*p* ≤ 0.05)

Analysis of variance revealed significant effects of pitaya variety on all physicochemical and biochemical parameters evaluated in the pulp (Supplementary Table 1), indicating substantial variability among the studied genotypes. Fruit weight differed significantly among varieties (Fig. 1A). The highest values were observed in ‘Golden de Israel’, ‘Costa Rica’, and ‘Physical Graffiti’, whereas ‘Connie Mayer’ exhibited the lowest fruit mass. These results are consistent with previous reports describing fruit weights ranging from 250 to 600 g for *Selenicereus costaricensis* and from 300 to 800 g for *S. undatus* [10]. Fruit mass is primarily determined by genetic factors, but agronomic practices and pollination efficiency also play a crucial role. Successful pollination promotes ovule fertilization and seed formation, which directly influences fruit size and weight in pitaya [11].

Significant variation was observed in pulp pH among varieties (Fig. 1B). ‘Costa Rica’ exhibited the highest pH values, followed by ‘Imperial’, ‘Nicarágua’, and ‘Connie Mayer’, whereas ‘Golden de Israel’ and ‘Purple Haze’ presented more acidic pulp. These values are comparable to those previously reported for *S. undatus* and *S. monacanthus*, with pH ranging between 4.8 and 5.4, reinforcing the mild acidity characteristic of pitaya fruits.Total soluble solids (TSS) content also varied significantly (Fig. 1C). ‘Connie Mayer’ showed the highest TSS (14.4 °Brix), followed by ‘Costa Rica’, ‘Nicarágua’, ‘Imperial’, and ‘Golden de Israel’, whereas ‘Purple Haze’ and ‘Physical Graffiti’ exhibited lower values. Similar TSS values have been reported for white- and red-fleshed pitayas, ranging from 13.2 to 13.7 °Brix [12]. In contrast, yellow-skinned pitayas (*S. megalanthus*) are known for much higher sugar contents (up to 22 °Brix), which explains their high consumer acceptance [9]. Titratable acidity (TA) differed significantly among varieties (Fig. 1D), with higher values observed in ‘Purple Haze’, ‘Physical Graffiti’, ‘Imperial’, and ‘Nicarágua’. Lower acidity was detected in ‘Costa Rica’ and ‘Connie Mayer’. According to [2], TA plays a key role in pitaya flavor perception, distinguishing sweet from sweet–acid cultivars. The relatively low acidity observed in all varieties explains the generally pleasant sensory profile of pitaya fruits.

**Fig. 2:**
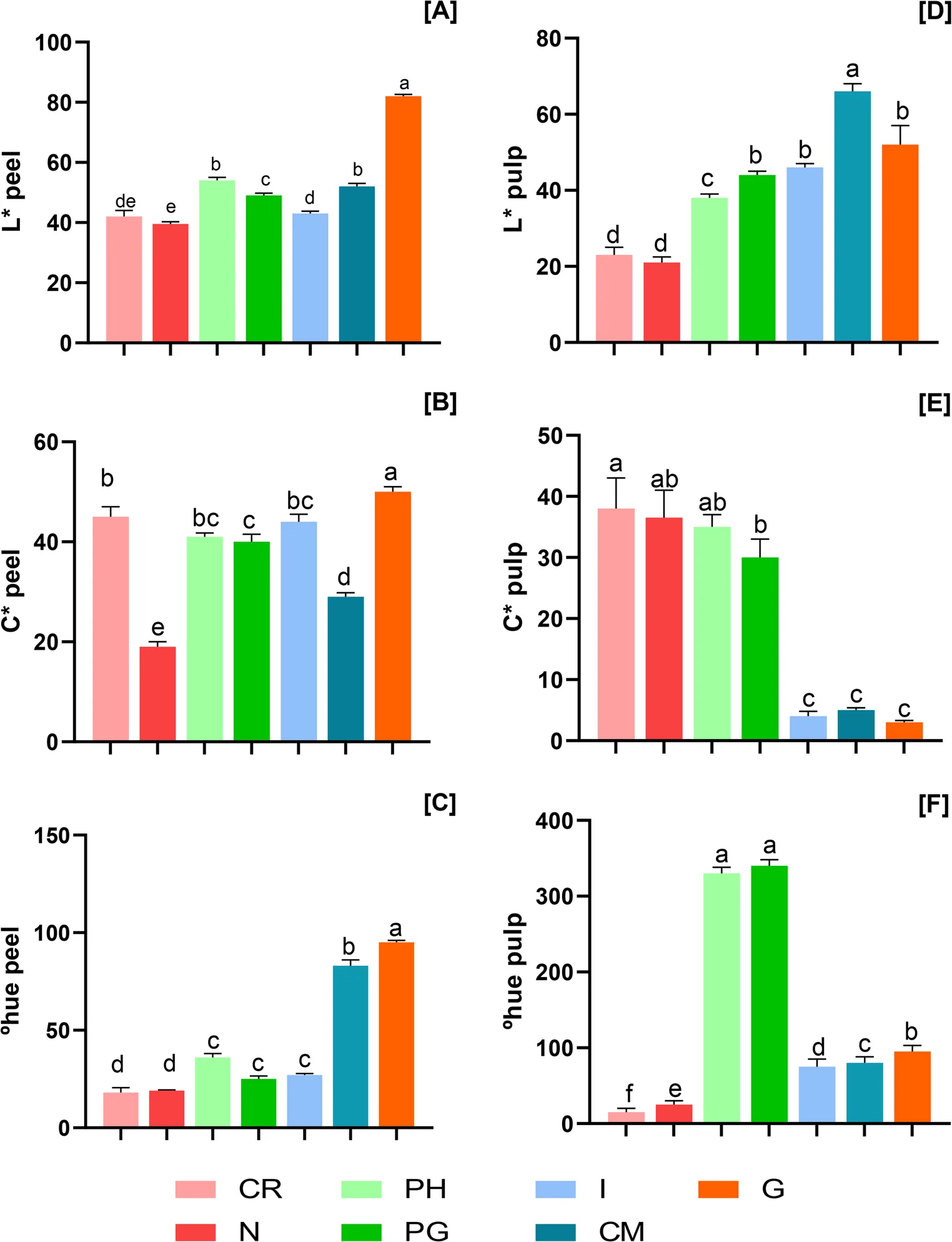
Color parameters. Lightness (L*) [**A**-**D**], Chroma (C*) [B-E], ºhue [**C**-**F**] of the pulp and peel of pitaya fruits of the varieties ‘Costa Rica’ (CR), ‘Nicaragua’ (N), ‘Purple Haze’ (PH), ‘Physical Graffiti’ (PG),‘Imperial’ (I), ‘Connie Mayer’ (CM), and ‘Golden de Israel’ (G). Letters above the bars indicate significant differences between varieties according to the LSD test (*p* ≤ 0.05)

The maturity index (TSS/TA ratio) was significantly higher in ‘Costa Rica’ and ‘Connie Mayer’, intermediate in ‘Nicarágua’, ‘Imperial’, and ‘Golden de Israel’, and lowest in ‘Purple Haze’ and ‘Physical Graffiti’ (Fig. 1E). This index reflects the balance between sugars and organic acids and is a key indicator of fruit palatability [13]. Reducing sugar content also varied significantly, with ‘Golden de Israel’ presenting the highest values, although not differing from ‘Connie Mayer’ (Fig. 1F). Similar carbohydrate profiles have been reported for *S. monacanthus* and *S. undatus*, with glucose and fructose as the predominant sugars [14]. Color parameters of peel and pulp clearly reflected varietal differences and pigment composition. Yellow-skinned ‘Golden de Israel’ exhibited the highest peel luminosity and chroma, whereas red-skinned varieties showed lower luminosity values (Fig. 2A-C). Pulp color analysis revealed a progressive increase in luminosity from red- to purple- and white-fleshed varieties, while chroma decreased accordingly (Fig. 2D-F). These results are consistent with previous studies linking pitaya flesh color to betalain concentration and composition [15].

### Bioactive Compounds and Antioxidant Activity

**Fig. 3:**
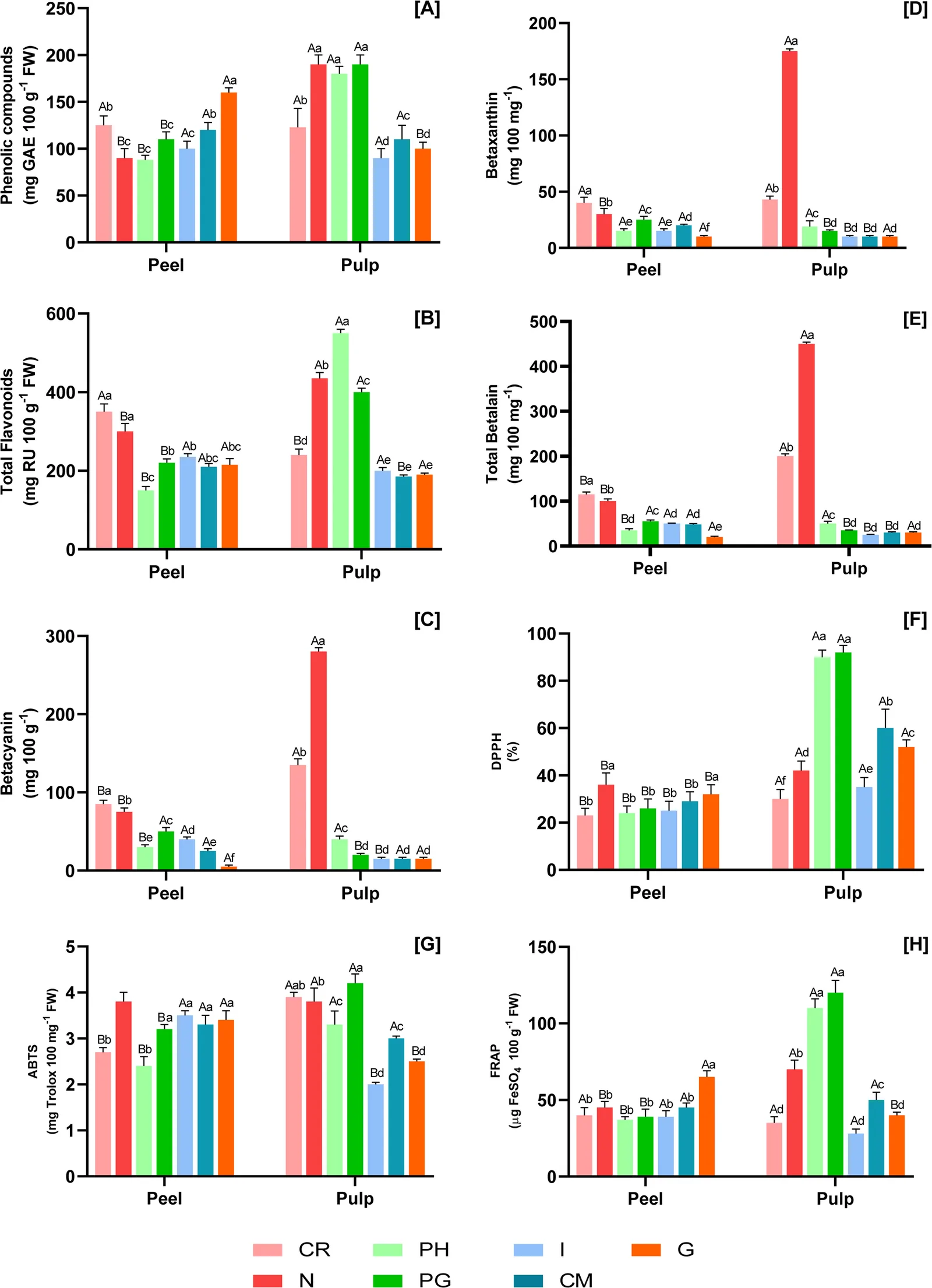
Bioactive compunds - Phenolic compounds [**A**], total flavonoids [**B**], Betacyanin [**C**], Betaxanthin [**D**], Total betalains [**E**] content and Antioxidant activity by the DPPH [**F**], ABTS+ [**G**], FRAP [**H**] from the peel and pulp of pitaya fruits of the varieties ‘Costa Rica’ (CR), ‘Nicaragua’ (N), ‘Purple Haze’ (PH), ‘Physical Graffiti’ (PG), ‘Imperial’ (I), ‘Connie Mayer’ (CM), and ‘Golden de Israel’ (G). Lowercase letters differentiate varieties and uppercase letters differentiate the peel and pulp of a single variety, according to the LSD test (*p* ≤ 0.05)

Significant differences in flavonoid, phenolic, betalain, and antioxidant profiles were observed among pitaya varieties and between peel and pulp tissues (Fig. 3). Significant differences in total phenolic compounds were observed among pitaya varieties and between peel and pulp tissues (Fig. 3A). In the peel, the highest phenolic content was detected in ‘Golden de Israel’ (approximately 160 mg GAE 100 g⁻¹ FW), followed by ‘Costa Rica’ (130 mg GAE 100 g⁻¹ FW), whereas ‘Nicarágua’ showed the lowest concentration (90 mg GAE 100 g⁻¹ FW). In the pulp, ‘Physical Graffiti’ and ‘Purple Haze’ exhibited the highest phenolic contents, reaching approximately 190 and 180 mg GAE 100 g⁻¹ FW, respectively, while ‘Imperial’ showed the lowest value (90 mg GAE 100 g⁻¹ FW). Previous studies have reported higher phenolic contents in red-fleshed pitayas compared to white-fleshed varieties [12], although the distribution between peel and pulp may vary among species [16].

Total flavonoid content also varied significantly among varieties and tissues (Fig. 3B). The highest flavonoid concentration in the peel was observed in ‘Costa Rica’ (approximately 350 mg RE 100 g⁻¹ FW), followed by ‘Nicarágua’ (300 mg RE 100 g⁻¹ FW), whereas ‘Purple Haze’ presented the lowest value (150 mg RE 100 g⁻¹ FW). In contrast, pulp tissues showed markedly higher flavonoid accumulation, particularly in ‘Purple Haze’ (550 mg RE 100 g⁻¹ FW), followed by ‘Nicarágua’ (440 mg RE 100 g⁻¹ FW) and ‘Physical Graffiti’ (400 mg RE 100 g⁻¹ FW). White-fleshed varieties, including ‘Imperial’, ‘Connie Mayer’, and ‘Golden de Israel’, exhibited considerably lower flavonoid contents, ranging from 180 to 220 mg RE 100 g⁻¹ FW. Similar variability between peel and pulp tissues has been previously reported in red-fleshed pitayas [17]. The occurrence of flavonoid-derived compounds such as ellagic acid arabinosides and quercetin glycosides in *S. monacanthus* reinforces the chemical diversity of these fruits [18].

Betacyanin concentrations differed markedly among varieties and tissues (Fig. 3C). Red-fleshed varieties showed the highest concentrations, particularly ‘Nicarágua’, which reached 280 mg 100 g⁻¹ FW in the pulp, followed by ‘Costa Rica’ with 135 mg 100 g⁻¹ FW. In the peel, the same varieties also exhibited elevated betacyanin levels, reaching approximately 80 and 90 mg 100 g⁻¹ FW, respectively. Conversely, white-fleshed varieties (‘Imperial’, ‘Connie Mayer’, and ‘Golden de Israel’) contained only trace amounts of these pigments. These findings confirm that betacyanins are the primary pigments responsible for the characteristic red coloration of pitaya pulp [19]. Significant variation was also observed for betaxanthins (Fig. 3D). The highest concentration was detected in the pulp of ‘Nicarágua’, reaching 175 mg 100 g⁻¹ FW, while ‘Costa Rica’ showed intermediate levels (45 mg 100 g⁻¹ FW). In the peel, betaxanthin concentrations ranged from approximately 10 to 45 mg 100 g⁻¹ FW, with the highest values observed in ‘Costa Rica’ and ‘Nicarágua’. White-fleshed varieties generally exhibited lower concentrations in both tissues. The marked predominance of betaxanthins in the pulp of ‘Nicarágua’ contributes significantly to its overall betalain profile. Total betalain content reflected the combined accumulation of betacyanins and betaxanthins (Fig. 3E). The highest concentration was observed in the pulp of ‘Nicarágua’, reaching approximately 450 mg 100 g⁻¹ FW, followed by ‘Costa Rica’ with 200 mg 100 g⁻¹ FW. In peel tissues, betalain contents were considerably lower but still substantial in red-fleshed varieties, ranging from approximately 100 to 120 mg 100 g⁻¹ FW. Purple- and white-fleshed varieties exhibited significantly lower betalain concentrations, generally below 60 mg 100 g⁻¹ FW. These results reinforce the importance of red-fleshed pitayas as natural sources of betalain pigments for functional food applications. corroborating previous reports [12].

Antioxidant activity (DPPH, FRAP and ABTS) varied markedly among varieties and tissues. Antioxidant activity determined by the DPPH assay varied substantially among varieties and tissues (Fig. 3F). The highest radical scavenging activity was observed in the pulp of ‘Physical Graffiti’ and ‘Purple Haze’, reaching approximately 92% and 90%, respectively. Intermediate values were recorded for ‘Connie Mayer’ (60%) and ‘Golden de Israel’ (52%), whereas ‘Costa Rica’ exhibited the lowest activity in the pulp (30%). In peel tissues, antioxidant activity ranged from approximately 24 to 36%. DPPH activity observed in purple-fleshed varieties is likely associated with their elevated phenolic and flavonoid concentrations. FRAP assay revealed pronounced differences in reducing power among pitaya varieties (Fig. 3H). The highest FRAP values were observed in the pulp of ‘Physical Graffiti’ and ‘Purple Haze’, reaching approximately 120 and 110 µg FeSO₄ 100 g⁻¹ FW, respectively. Intermediate values were found in ‘Nicarágua’ (70 µg FeSO₄ 100 g⁻¹ FW) and ‘Connie Mayer’ (50 µg FeSO₄ 100 g⁻¹ FW), whereas ‘Imperial’ exhibited the lowest reducing power (approximately 28 µg FeSO₄ 100 g⁻¹ FW). These results further support the superior antioxidant potential of purple-fleshed varieties. Because FRAP measures the electron-donating capacity of antioxidants, the elevated values observed in ‘Physical Graffiti’ and ‘Purple Haze’ are likely associated with their high concentrations of phenolic compounds and flavonoids. Similar trends were observed for ABTS radical scavenging activity (Fig. 3G). Highest value was detected in the pulp of ‘Physical Graffiti’ (4.2 mg Trolox 100 g⁻¹ FW), followed by ‘Costa Rica’ and ‘Nicarágua’ (approximately 3.9 mg Trolox 100 g⁻¹ FW). Lowest value was recorded in ‘Imperial’ (2.0 mg Trolox 100 g⁻¹ FW). Peel tissues generally exhibited values between 2.5 and 3.8 mg Trolox 100 g⁻¹ FW. ABTS assay evaluates both hydrophilic and lipophilic antioxidant compounds, which may explain the strong responses observed in varieties containing distinct classes of bioactive metabolites [20].

Differences among DPPH, ABTS, and FRAP responses are expected because each assay is based on distinct reaction mechanisms. The DPPH method primarily evaluates the ability of antioxidants to donate hydrogen atoms to a stable free radical, whereas ABTS measures both hydrophilic and lipophilic antioxidant compounds through electron-transfer reactions. In contrast, FRAP estimates the reducing power of antioxidants by quantifying their capacity to reduce ferric ions (Fe³⁺) to ferrous ions (Fe²⁺). Consequently, differences in the relative abundance of phenolic compounds, flavonoids, betalains, and other antioxidant metabolites among pitaya varieties may result in distinct responses depending on the assay employed. Similar assay-dependent variations have been reported in previous studies evaluating antioxidant activity in pitaya and other tropical fruits.

Principal component analysis (PCA) efficiently discriminated pitaya varieties according to biochemical composition and color parameters (Supplementary Figs. 2 and 3). In peel tissues, PC1 and PC2 explained 78.47% of the total variance and separated red-fleshed varieties (CR and N), associated with higher betalain and flavonoid contents, from CM and G, which were associated with higher luminosity, chroma, and FRAP values. In pulp tissues, the first two components explained 78.36% of total variance and clearly separated white-, red-, and purple-fleshed varieties according to pigment composition, antioxidant activity, and color parameters. Similar associations between antioxidant activity, phenolic compounds, and betalains have been previously reported in pitaya fruits [21]. Hierarchical cluster analysis confirmed the marked biochemical heterogeneity among varieties and between peel and pulp tissues (Supplementary Fig. 4). Purple-fleshed varieties (PH and PG) clustered according to their higher phenolic contents and antioxidant activity, whereas the yellow peel variety G formed an isolated group associated with higher luminosity and lower betalain contents. Similar clustering patterns have been reported for *S. megalanthus* due to its distinct peel coloration and biochemical profile [22].

### Mineral Composition and Nutritional Relevance

Mineral composition varied significantly between peel and pulp tissues and among pitaya varieties (Tables 1 and 2). Higher concentrations of P, S, and N were generally observed in the pulp, whereas peel tissues showed higher levels of Mg, Ca, and K. Potassium was the predominant macronutrient in all varieties, particularly in the peels of PH and PG, corroborating previous reports for *S. monacanthus* and other Selenicereus species [23]. Because potassium intake is associated with cardiovascular health benefits, pitaya fruits and by-products may represent valuable dietary sources of this mineral. Calcium, phosphorus, and magnesium also occurred at nutritionally relevant concentrations, while iron was the predominant micronutrient in several varieties, especially in Imperial pulp, consistent with previous findings for *S. setaceus* [20]. The combination of minerals with bioactive compounds such as betalains and phenolics may contribute to the biological properties previously reported for pitaya, including antihemolytic, antianemic, and antibacterial activities [24]. From an agronomic perspective, the predominance of potassium agrees with nutrient export patterns previously described for *S. undatus* and *S. monacanthus* [25].

**Table 1:** Plant macronutrients in the peel and pulp of pitaya varieties

	N	P	K	Ca	Mg	S
			g kg-1			
			**Peel**			
**CR**	8.0±0.48^Bb^	2.9±0.23^Bab^	60.0±4.20^Abc^	9.0±0.81^Ab^	4.9±0.25^Ac^	0.9±0.05^Bbc^
**N**	9.0±0.27^Bb^	2.7±0.16^Bb^	59.1±2.36^Abc^	9.1±0.45^Ab^	4.8±0.14^Acd^	1.0±0.06^Bb^
**PH**	6.3±0.35^Bc^	2.2±0.12^Bc^	73.6±4.05^Aa^	8.3±0.27^Ac^	5.3±0.29^Ab^	0.9±0.04^Bc^
**PG**	10.2±0.62^Ba^	3.1±0.19^Ba^	76.2±4.57^Aa^	10.1±0.30^Aa^	7.1±0.43^Aa^	1.0±0.02^Bb^
**I**	8.0±0.24^Bb^	2.8±0.08^Bb^	61.1±2.44^Ab^	8.0±0.24^Ac^	4.7±0.24^Acd^	1.2±0.04^Ba^
**CM**	10.4±0.21^Ba^	2.8±0.06^Bb^	56.5±1.13^Ac^	10.1±0.51^Aa^	4.5±0.09^Ade^	1.3±0.05^Ba^
**G**	10.3±0.31^Ba^	2.1±0.06^Bc^	50.5±2.53^Ad^	8.0±0.24^Ac^	4.2±0.29^Ae^	1.2±0.04^Ba^
			**Pulp**			
**CR**	20.1±0.80^Ad^	3.3±0.13^Ac^	20.1±1.20^Bcd^	2.0±0.08^Bab^	2.7±0.14^Bde^	1.8±0.07^Abc^
**N**	22.2±1.32^Ab^	3.0±0.18^Ad^	25.0±1.13^Bab^	2.1±0.12^Bab^	3.0±0.21^Bcd^	1.9±0.10^Ab^
**PH**	21.8±0.96^Abc^	4.1±0.18^Aa^	27.2±1.50^Ba^	1.8±0.08^Bab^	3.7±0.13^Ba^	1.9±0.08^Ab^
**PG**	24.6±0.99^Aa^	3.9±0.16^Aa^	24.2±0.97^Bab^	1.8±0.07^Bab^	3.5±0.07^Bab^	2.1±0.08^Aa^
**I**	21.0±1.26^Abcd^	3.2±0.19^Acd^	21.0±0.42^Bbcd^	2.1±0.12^Bab^	3.2±0.10^Bbc^	1.7±0.10^Acd^
**CM**	21.1±1.26^Abcd^	3.6±0.11^Ab^	23.5±1.41^Babc^	2.3±0.05^Ba^	2.9±0.17^Bcd^	1.7±0.10^Ac^
**G**	20.4±0.82^Acd^	3.3±0.07^Ac^	19.4±0.78^Bd^	1.7±0.07^Bb^	2.5±0.18^Be^	1.6±0.06^Ad^

Capital letters differentiate the parts of the fruit (peel and pulp) of a single variety and lowercase letters differentiate the varieties in a single part of the fruit (peel or pulp). according to the LSD test (*p* ≤ 0.05). ‘Costa Rica’ (CR), ‘Nicaragua’ (N), ‘Purple Haze’ (PH), ‘Physical Graffiti’ (PG), ‘Imperial’ (I), ‘Connie Mayer’ (CM), and ‘Golden de Israel’ (G)

**Table 2:** Plant micronutrients from the skin and pulp of pitaya varieties

	B	Cu	Fe	Mn	Zn
			mg kg-1		
			**Peel**		
**CR **	128.0±8.96^Aab^	10.0±0.60^Aa^	69.0±2.76^Ab^	79.0±4.74^Ab^	41.0±3.28^Bcd^
**N **	122.0±3.66^Ab^	10.0±0.70^Ba^	44.0±1.32^Bd^	70.0±2.80^Ac^	55.0±2.75^Aa^
**PH**	100.5±5.53^Ad^	8.0±0.44^Bc^	65.0±5.01^Bb^	84.0±3.70^Aa^	39.0±2.15^Bd^
**PG**	131.7±7.90^Aa^	10.0±0.60^Aa^	40.0±2.80^Bd^	73.0±4.38^Ac^	57.0±2.85^Aa^
**I **	109.1±3.27^Ac^	9.0±0.27^Ab^	43.0±3.01^Bd^	65.0±1.95^Ad^	53.0±1.06^Aab^
**CM**	134.6±2.69^Aa^	9.0±0.45^Bb^	127.0±2.54^Ba^	58.0±3.19^Ae^	44.0±0.88^Ac^
**G**	82.0±4.92^Ae^	9.0±0.27^Ab^	50.0±0.75^Bc^	63.0±1.89^Ad^	50.0±1.50^Bb^
			**Pulp**		
**CR**	68.9±4.83^Ba^	8.0±0.24^Bd^	74.0±2.96^Ac^	26.0±1.30^Ba^	49.0±2.94^Aa^
**N**	50.1±1.80^Bc^	11.0±0.66^Ab^	59.0±2.12^Ad^	22.0±1.32^Babc^	44.0±3.34^Bbc^
**PH**	36.6±0.88^Be^	12.0±0.53^Aa^	78.0±4.21^Ac^	23.0±1.01^Bab^	47.0±1.60^Aab^
**PG**	44.8±1.79^Bcd^	9.0±0.36^Bc^	62.0±4.96^Ad^	19.0±0.76^Bbc^	47.0±2.82^Bab^
**I**	40.9±3.28^Bde^	9.0±0.54^Ac^	75.0±0.75^Ac^	19.0±1.14^Bbc^	42.0±0.42^Bc^
**CM**	61.0±3.66^Bb^	11.0±0.66^Ab^	160.0±3.20^Ab^	20.0±1.20^Bbc^	41.0±2.46^Ac^
**G**	34.9±1.39^Be^	8.0±0.44^Bd^	175.0±7.00^Aa^	18.0±0.72^Bc^	45.0±2.97^Aabc^

Capital letters differentiate the parts of the fruit (peel and pulp) of a single variety. Lowercase letters differentiate the varieties in a single part of the fruit (skin or pulp). by the LSD test (p ≤ 0.05). ‘Costa Rica’ (CR), ‘Nicaragua’ (N), ‘Purple Haze’ (PH), ‘Physical Graffiti’ (PG), ‘Imperial’ (I), ‘Connie Mayer’ (CM), and ‘Golden de Israel’ (G)

Principal component analysis (PCA) of mineral composition clearly separated peel and pulp tissues (Supplementary Fig. 5), with the first two components explaining 80.21% of total variance. Pulp tissues were associated mainly with higher P, S, N, and Fe contents, whereas peel tissues were associated with higher Mg, Ca, K, B, and Mn concentrations. In addition, PH, PG, and N varieties showed distinct nutritional profiles compared with the remaining genotypes. Hierarchical cluster analysis confirmed the nutritional heterogeneity among varieties and tissues (Supplementary Fig. 6), revealing clear grouping patterns between peel and pulp samples. These results highlight the substantial nutritional diversity among pitaya genotypes and reinforce their potential for selection in breeding programs and functional food applications [22].

## Conclusion

Based on the results, it can be concluded that pitaya fruits from the studied varieties exhibit important physicochemical, biochemical, and nutritional characteristics for fresh consumption, particularly notable for their high maturity index resulting primarily from low acidity content and moderate soluble solids content, which provides an excellent flavor profile. Fruits from red-fleshed varieties, Costa Rica and Nicarágua, possess higher betalain contents, whereas purple-fleshed varieties, Purple Haze and Physical Graffiti, present higher contents of phenolic compounds, flavonoids, and greater antioxidant activity. Both pulp and peel from pitaya varieties exhibit excellent mineral profiles for human consumption, with significant values of potassium and iron, particularly in the peels of Physical Graffiti, which showed the highest macronutrient content, and Connie Mayer, which presented the highest micronutrient content. Thus, the inclusion of these fruits in dietary patterns can contribute to increased intake of minerals and bioactive antioxidant compounds. The processing residue (peel) also demonstrates potential as a raw material for the extraction of functional compounds of interest.

## Supplementary Information

Below is the link to the electronic supplementary material.

Supplementary File 1 (DOCX 1.25 MB)

## Data Availability

No datasets were generated or analysed during the current study.
