# Supplementary material for: Bioactive Compounds, Antioxidant Capacity and Mineral Profile of Pulp and Peel from Diverse Selenicereus (Pitaya) Varieties in Brazil
Source: Plant Foods Hum Nutr. 2026 Jul 30;81(3):103. doi: 10.1007/s11130-026-01553-6 (PMC13421253; doi:10.1007/s11130-026-01553-6)
Supplement: Supplementary file 1 — (DOCX 1.25 MB) [file 11130_2026_1553_MOESM1_ESM.docx]

*Supplemental material*

# Bioactive compounds, antioxidant capacity and mineral profile of pulp and peel from diverse *Selenicereus* (pitaya) varieties in Brazil

**Rogerio Lopes Vieites^1^. Andres Felipe Gaona Acevedo^1^**

## Materials and Methods

### Plant Material

Pitaya fruits from seven varieties (Supplementary Fig. 1)—‘Costa Rica’ (Selenicereus costaricensis), ‘Nicarágua’ (*Selenicereus guatemalensis*), ‘Purple Haze’ and ‘Physical Graffiti’ (Selenicereus guatemalensis × Selenicereus undatus), ‘Imperial’ (*Selenicereus setaceus*), ‘Connie Mayer’ (*Selenicereus stenopterus* × *Selenicereus undatus*), and ‘Golden de Israel’ (*Selenicereus undatus*)—were harvested from a commercial orchard in Indaiatuba, São Paulo, Brazil (23°05′S, 47°13′W). All plants were cultivated under the same agronomic conditions. Fruits were harvested between 34 and 38 days after anthesis when they reached commercial maturity, characterized by complete peel coloration, full development of bract pigmentation, and absence of green areas on the fruit surface, according to maturity indicators previously described [1], and transported to the Fruit and Vegetable Postharvest Laboratory, São Paulo State University (UNESP), Botucatu, Brazil. For each variety, fifteen fruits were harvested and divided into five biological replicates, each replicate consisting of three fruits. All physicochemical, biochemical, and mineral analyses were performed using these biological replicates. After harvest, fruits were washed and manually peeled using stainless-steel knives. Peel and pulp tissues were carefully separated, avoiding cross-contamination between tissues, and subsequently processed for biochemical and mineral analyses.

### Fruit weight and color of peel and pulp

Fruit weight was determined using a semi-analytical balance (AMEL 334-B, Milano, Italy). Peel color was measured at three points per fruit (equatorial region and two opposite ends), while pulp color was evaluated at three random points after longitudinal sectioning of the fruits. Color measurements were performed using a Minolta CR400 colorimeter according to the CIELAB system (L*, a*, b*). Hue angle (h°) and chroma (C*) were calculated from a* and b* values to characterize color intensity and tonality of peel and pulp tissues.

### Physicochemical properties analysis

#### Determination of pH, Soluble Solids (SS), titratable acidity (TA), ripe index (SS / TA ) and reducing sugars (RS).

Pulp pH was measured using a digital potentiometer (Consort P407, SchottGerate, Belgium) according to IAL [2] . Soluble solids (SS) were determined by refractometry (model RT-30 ATC, Japan) and expressed as °Brix. Titratable acidity (TA) was determined by titration with 0.1 N NaOH and expressed as % malic acid. The maturity index was calculated as the SS/TA ratio. Reducing sugars were determined according to the Somogyi–Nelson method [3], using spectrophotometric readings at 535 nm, and results were expressed as percentage (%).

### Bioactive compounds and antioxidant activity

#### Preparation of acetone extract from fruit

A mixture of acetone and water (80:20 v/v) was used for extraction. Fruit extracts were obtained in triplicate. 1 gram of pitaya fruit pulp was weighed into Falcon tubes, to which 10 mL of the acetone:water mixture (80:20 v/v) was added. The acetone:water (80:20, v/v) mixture was selected because of its high extraction efficiency for phenolic compounds and other antioxidant metabolites from plant tissues, providing a broad recovery of both polar and moderately non-polar compounds. The tubes containing the pitaya fruit pulp and acetone solvent were homogenized with a Ultra-Turrax for a few minutes at room temperature. The extracts were then centrifuged at 4,500 ×g for 15 min at room temperature. The supernatant was removed and stored in dark bottles at 8°C until analysis of total phenolic compounds and antioxidant activity by the DPPH method.

#### Determination of total phenolic compounds, total flavonoids and betalain content

Total phenolic compounds were determined in acetone extracts using the Folin–Ciocalteu spectrophotometric method [4]. Absorbance was measured at 740 nm, and results were expressed as mg gallic acid equivalents per 100 g fresh weight (mg GAE 100 g⁻¹ FW) based on a gallic acid standard curve. Quantification was performed using a gallic acid calibration curve (0–100 mg L⁻¹; R² > 0.99). Total flavonoids were determined by the colorimetric method described by [5]. Absorbance was measured at 510 nm, and results were expressed as mg rutin equivalents per 100 g fresh weight (mg RE 100 g⁻¹ FW) using a rutin calibration curve. Flavonoid concentration was determined using a rutin standard curve (0–100 mg L⁻¹; R² > 0.99). All analyses were performed in triplicate. Betalain extraction and quantification were performed according to [6]. Fresh samples were extracted with 80% aqueous methanol, and absorbance was measured at 538 nm (betacyanins) and 483 nm (betaxanthins). Pigment contents were calculated using their respective molecular weights and molar extinction coefficients and expressed as mg 100 g⁻¹ fresh weight. All analyses were performed in triplicate.

#### Antioxidant Activity by the DPPH method, ABTS^+^ radical scavening and FRAP method.

Antioxidant activity was determined using the DPPH radical scavenging assay according to [7]. Absorbance was measured at 517 nm after incubation in the dark, and results were expressed as percentage of radical scavenging activity. ABTS radical scavenging activity was determined according to [8]. The ABTS⁺ solution was adjusted to an absorbance of 0.70 ± 0.02 at 732 nm prior to analysis. Results were expressed as mg Trolox equivalents per 100 g fresh weight (mg Trolox 100 g⁻¹ FW). Ferric reducing antioxidant power (FRAP) was determined according to [9]. Absorbance was measured at 593 nm, and results were expressed as μg FeSO₄ 100 g⁻¹ fresh weight.

### Mineral Composition Determination

Nutrient contents were determined separately in peel and pulp samples. Tissues were dried at 65 °C for 72 h, ground, and analyzed for mineral composition. All mineral concentrations are expressed on a dry weight basis (DW) for N, P, K, Ca, Mg, S, B, Cu, Fe, Zn, and Mn according to [10]. Results were expressed as g kg⁻¹ for macronutrients and mg kg⁻¹ for micronutrients.

### Statistical Analysis

Data were subjected to the Shapiro–Wilk normality test followed by one-way ANOVA for physicochemical characterization and two-way ANOVA (2 × 7) for biochemical and nutritional analyses of peel and pulp tissues. Means were compared using the LSD test at p ≤ 0.05 and p ≤ 0.01. Analyses were performed using Sisvar 5.6 and GraphPad Prism 8. Multivariate analyses included hierarchical cluster analysis using Ward’s method and Euclidean distance, as well as principal component analysis (PCA) performed in JMP 10 (SAS Institute Inc., USA).

## Reference

1. Magalhães DS, da Silva DM, Ramos JD, et al (2019) Changes in the physical and physico-chemical characteristics of red-pulp dragon fruit during its development. Sci Hortic (Amsterdam) 253:180–186. https://doi.org/10.1016/j.scienta.2019.04.050

2. IAL (2008) Metodos fisicos e quimicas para analise de alimento., 4th ed. Instituto Adolfo Lutz, Sao Paulo

3. Nelson N (1944) A photometric adaptation of the somogyi method for the determinnation of glucose. J Biol Chem 03:375–380

4. Singleton V, Orthofer R, Lamuela Raventos R (1999) Analysis of total phenols ad other oxidation subtrates and antioxidants by means of folin-ciocalteu reagent. In: Methods of Enzimology. pp 152–178

5. Zhishen J, Mengcheng T, Jianming W (1999) The determination of flavonoid contents in mulberry and their scavenging effects on superoxide radicals. Food Chem 64:555–559. https://doi.org/10.1016/S0308-8146(98)00102-2

6. García-Cruz L, Valle-Guadarrama S, Salinas-Moreno Y, Joaquín-Cruz E (2013) Physical, Chemical, and Antioxidant Activity Characterization of Pitaya (Stenocereus pruinosus) Fruits. Plant Foods Hum Nutr 68:403–410. https://doi.org/10.1007/s11130-013-0391-8

7. Brand-Williams W, Cuvelier ME, Berset C (1995) Use of a Free Radical Method to Evaluate Antioxidant Activity. Leb u-Technol 28:25–30. https://doi.org/10.1016/S0023-6438(95)80008-5

8. Re R, Pellegrini N, Proteggente A, et al (1999) Antioxidant Activity Applying an Improved Abts Radical. Free Radic Biol Med 26:1231–1237. https://doi.org/10.1016/S0891-5849(98)00315-3

9. Benzie IFF, Strain JJ (1996) Ferric reducing (antioxidant) power as a measure of antioxidant capacity: the FRAP assay. Anal Biochem 239:70–76

10. Malavolta E, Vitti GC, Oliveira SA (1997) Avaliação do estado nutricional das plantas: princípios e aplicações, 2nd ed. Associação Brasileira para Pesquisa da Potassa e do Fosfato, Piracicaba

**
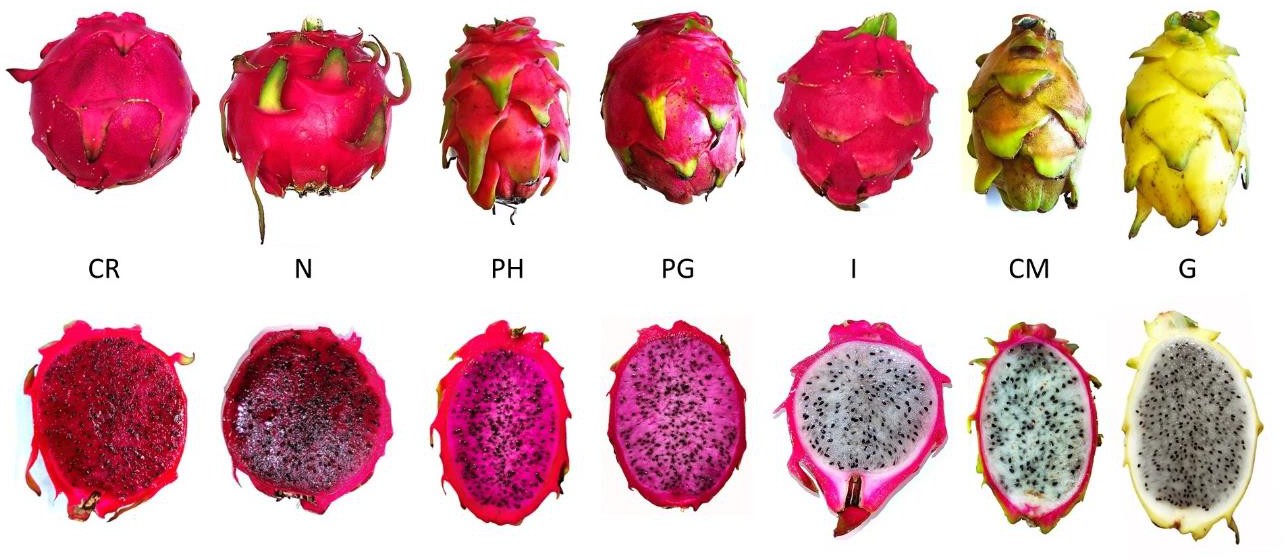
**

**Supplementary Fig. 1.** External and internal images of the pitaya varieties used in the physical-chemical, biochemical, and nutritional characterization. External and internal images after longitudinal cutting of pitaya fruits of the varieties ‘Costa Rica’ “Roxa do Pará” (CR), 'Nicaragua' “Orejona” (N), ‘Purple Haze’ (PH), ‘Physical Graffiti’ (PG), ‘Imperial’ (I), ‘Connie Mayer’ (CM), and ‘Golden de Israel’ (G).

| 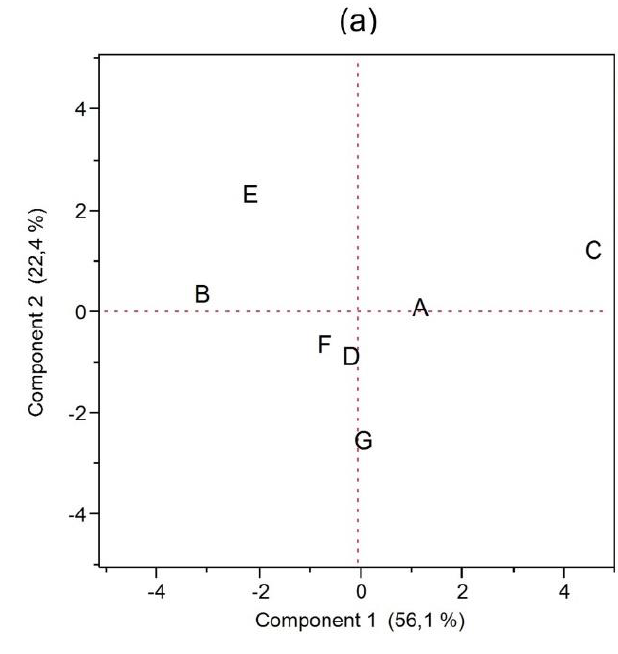 | 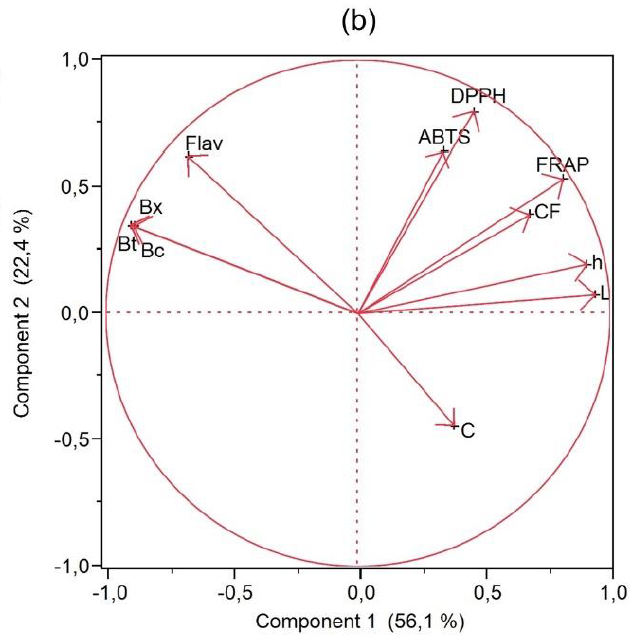 |
| --- | --- |
| **Supplementary Fig. 2.** Principal component analysis of eleven biochemical analyses and peel color of seven varieties of pitaya fruit. Score chart (a): ‘Costa Rica’ (B), ‘Nicaragua’ (E), ‘Purple Haze’ (G), ‘Physical Graffiti’ (F), ‘Imperial’ (D), ‘Connie Mayer’ (A) and ‘Golden de Israel’ (C) Load matrix graph (b): L (lightness), C (chroma), h (°hue), Bt (Betalaínas), Bc (Betacianina), Bx (Betaxantina), Flav (Flavonoids), CF (Phenolic compounds), DPPH (Antioxidant activity), ABTS (Radical scavenging activity), FRAP. | |

| 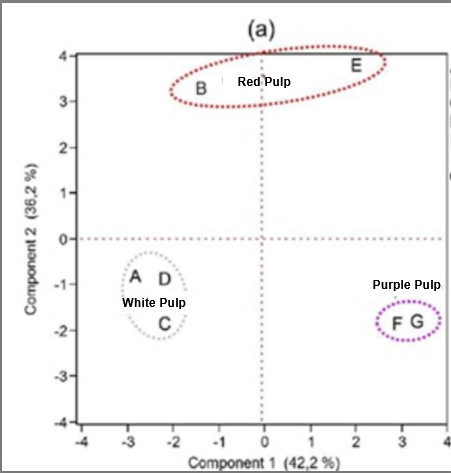 | 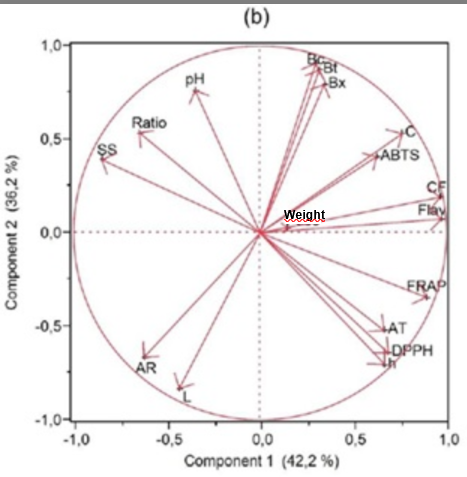 |
| --- | --- |
| **Supplementary Fig. 3.** Principal component analysis of seventeen physicochemical, biochemical, and color analyses of the pulp of seven varieties of pitaya fruit. Score chart (a): ‘Costa Rica’ (B), ‘Nicaragua’ (E), ‘Purple Haze’ (G), ‘Physical Graffiti’ (F), ‘Imperial’ (D), ‘Connie Mayer’ (A), and ‘Golden de Israel’ (C). Load matrix graph (b): Weight (fruit weight), pH (hydrogen ion potential), SS (soluble solids), TA (titratable acidity), ratio (ripeness index), AR (reducing sugar), L (lightness), C (chroma), h (°hue), Bt (betalains), Bc (betacyanin), Bx (betaxanthin), Flav (flavonoids), CF (phenolic compounds), DPPH (antioxidant activity), ABTS (radical scavenging activity), FRAP (reducing capacity). | |


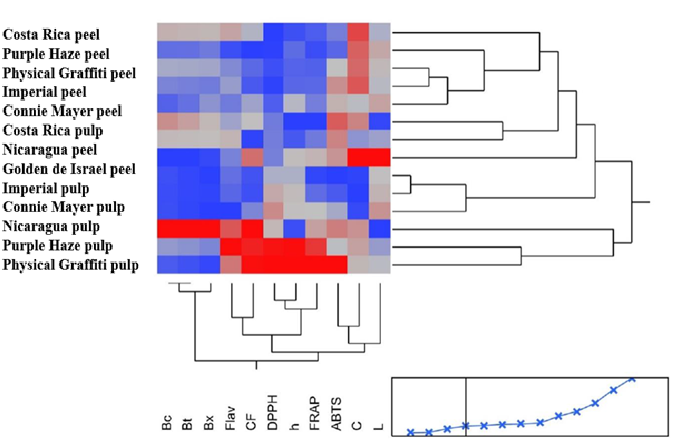


**Supplementary Fig. 4*.***  Two-way hierarchical cluster analysis of the eleven biochemical analyses and color of the skin and pulp of seven varieties of pitaya fruit, generated by the Ward method. Analyses performed: L (lightness), C (chroma), h (°hue), Bt (betalains), Bc (betacyanin), Bx (betaxanthin), Flav (flavonoids), CF (phenolic compounds), DPPH (antioxidant activity), ABTS (radical scavenging activity), FRAP (reducing capacity). Blue represents lower values in the analyses and red represents higher values.

| 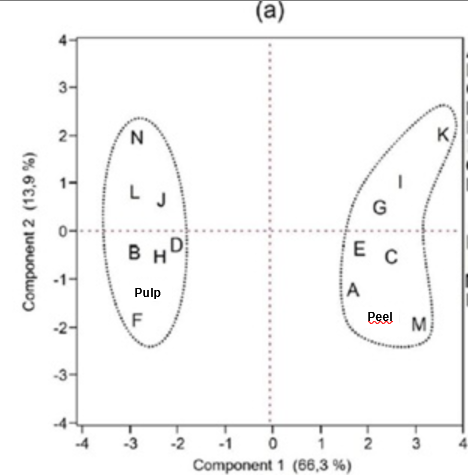 | 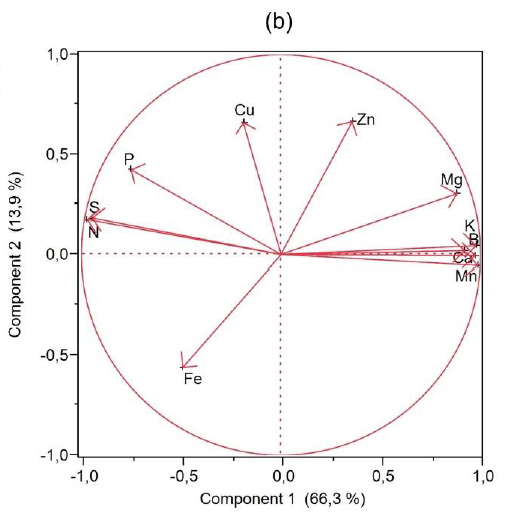 |
| --- | --- |
| **Supplementary Fig. 5.** Principal component analysis of mineral nutrients (N, P, K, Ca, Mg, S, B, Cu, Fe, Zn, and Mn) in the skin and pulp of pitaya fruit of the varieties ‘Costa Rica’ (CR), ‘Nicaragua’ (N), ‘Purple Haze’ (PH), ‘Physical Graffiti’ (PG), ‘Imperial’ (I), ‘Connie Mayer’ (CM), and ‘Golden de Israel’ (G). Score chart (a): ‘Connie Mayer’ peel (A), ‘Connie Mayer’ pulp (B) ‘Costa Rica’ skin (C), ‘Costa Rica’ pulp (D), ‘Golden de Israel’ peel (E), ‘Golden de Israel’ pulp (F), ‘Imperial’ peel (G), ‘Imperial’ pulp (H), ‘Nicaragua’ peel (I), ‘Nicaragua’ pulp (J), ‘Physical Graffiti’ peel (K), ‘Physical Graffiti’ pulp (L), ‘Purple Haze’ peel (M), and ‘Purple Haze’ pulp (N). Load matrix graph (b): N, P, K, Ca, Mg, S, B, Cu, Fe, Zn, and Mn | |


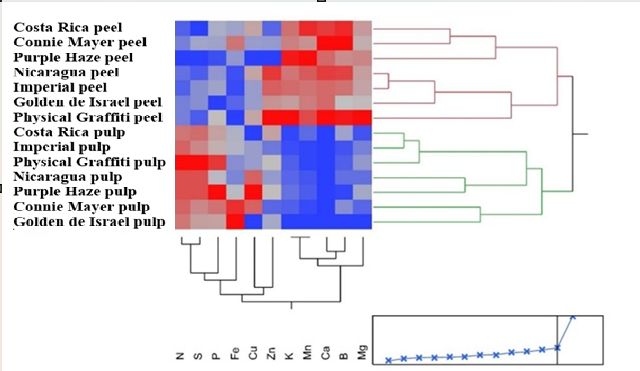


**Supplementary Fig. 6.** Two-way hierarchical clustering of the eleven mineral nutrients in the skin and pulp of seven varieties of pitaya fruit, generated using the Ward method. Mineral nutrients analyzed: N, P, K, Ca, Mg, S, B, Cu, Fe, Zn, and Mn. Blue represents lower values in the analyses and red represents higher values.

**Supplementary Table 1**. Analysis of variance (One-Way and Two-Way ANOVA) of pitaya fruit varieties fruit parts (peel and pulp) and the interaction between these factors through physical-chemical characterization analyses of the pulp and biochemical and nutritional analyses of the peel and pulp of the fruit.

|  | **Variety (V)** | | **Part (P)  (peel and pulp)** | | **V x P** | |
| --- | --- | --- | --- | --- | --- | --- |
| **Parameters** | |  |  |  |  |  |
|  | **Fc** | ***p*** | **Fc** | ***p*** | **Fc** | ***p*** |
| Fruit weight(g) | 27.283 | 0.000** | - | - | - | - |
| pH | 182.267 | 0.000** | - | - | - | - |
| Soluble solids (°Brix) | 17.856 | 0.000** | - | - | - | - |
| Titratable acidity (% TA) | 22.394 | 0.000** | - | - | - | - |
| *ratio* (SS/AT) | 64.996 | 0.000** | - | - | - | - |
| Reducing sugar (%) | 10.738 | 0.000** | - | - | - | - |
| Lightness peel | 174.483 | 0.000** | - | - | - | - |
| Chroma peel | 89.662 | 0.000** | - | - | - | - |
| °hue peel | 344.725 | 0.000** | - | - | - | - |
| Lightness pulp | 52.960 | 0.000** | - | - | - | - |
| Chroma pulp | 92.245 | 0.000** | - | - | - | - |
| °hue pulp | 3865.270 | 0.000** | - | - | - | - |
| Total Betalaíns (mg/100g) | 2050.771 | 0.000** | 877.996 | 0.000** | 1064.515 | 0.000* |
| Betacyanin (mg/100g) | 1486.510 | 0.000** | 722.818 | 0.000** | 663.609 | 0.000** |
| Betaxanthin (mg/100g) | 2555.901 | 0.000** | 804.101 | 0.000** | 1703.872 | 0.000** |
| Total Flavonoids (mg RU/100g) | 52.891 | 0.000** | 82.105 | 0.000** | 63.016 | 0.000** |
| Phenolic compounds (mg/100g) | 36.454 | 0.000** | 191.140 | 0.000** | 122.328 | 0.000** |
| Antioxidant activity (% DPPH) | 137.285 | 0.000** | 1776.957 | 0.000** | 151.991 | 0.000** |
| ABTS^+^ (mg Trolox/100g) | 9.282 | 0.000** | 10.061 | 0.026* | 18.730 | 0.000** |
| FRAP (µg FeSO4/100g) | 25.038 | 0.000** | 89.867 | 0.000** | 36.356 | 0.000** |
| N (mg/100g) | 13.741 | 0.000** | 2575.201 | 0.000** | 8.295 | 0.000** |
| P (mg/100g) | 17.293 | 0.000** | 333.724 | 0.000** | 21.924 | 0.000** |
| K (mg/100g) | 32.744 | 0.000** | 2771.597 | 0.000** | 14.694 | 0.000** |
| Ca (mg/100g) | 14.865 | 0.000** | 4950.568 | 0.000** | 9.827 | 0.000** |
| Mg (mg/100g) | 53.070 | 0.000** | 902.662 | 0.000** | 17.731 | 0.000** |
| S (mg/100g) | 5.772 | 0.000** | 1288.976 | 0.000** | 26.156 | 0.000** |
| B (mg/100g) | 66.794 | 0.000** | 2327.315 | 0.000** | 11.407 | 0.000** |
| Cu (mg/100g) | 11.744 | 0.000** | 7.549 | 0.010** | 25.165 | 0.000** |
| Fe (mg/100g) | 590.533 | 0.000** | 1036.623 | 0.000** | 202.820 | 0.000** |
| Mn (mg/100g) | 30.330 | 0.000** | 4011.141 | 0.000** | 13.031 | 0.000** |
| Zn (mg/100g) | 12.412 | 0.000** | 21.458 | 0.000** | 18.320 | 0.000** |

* ns: represents not significant; * represent p ≤ 0.05 and ** represent p ≤ 0.01
